# A Fast Robot Identification and Mapping Algorithm Based on Kinect Sensor

**DOI:** 10.3390/s150819937

**Published:** 2015-08-14

**Authors:** Liang Zhang, Peiyi Shen, Guangming Zhu, Wei Wei, Houbing Song

**Affiliations:** 1 National School of Software, Xidian University, Xi’an 710071, China; E-Mails: liangzhang@xidian.edu.cn (L.Z.); pyshen@xidian.edu.cn (P.S.); gmzhu@xidian.edu.cn (G.Z.); 2 School of Computer Science and Engineering, Xi’an University of Technology, 5 Jinhua S. Rd., Xi’an 710048, China; E-Mail: weiwei@xaut.edu.cn; 3 State Key Laboratory for Strength and Vibration of Mechanical Structures, Xi’an Jiaotong University, 28 Xianning W. Rd., Xi’an 710049, China; 4 Department of Electrical and Computer Engineering, West Virginia University, 405 Fayette Pike, Montgomery, WV 25136, USA

**Keywords:** SLAM, Kinect, RGB-D SLAM, improved algorithm, Dr Robot X80

## Abstract

Internet of Things (IoT) is driving innovation in an ever-growing set of application domains such as intelligent processing for autonomous robots. For an autonomous robot, one grand challenge is how to sense its surrounding environment effectively. The Simultaneous Localization and Mapping with RGB-D Kinect camera sensor on robot, called RGB-D SLAM, has been developed for this purpose but some technical challenges must be addressed. Firstly, the efficiency of the algorithm cannot satisfy real-time requirements; secondly, the accuracy of the algorithm is unacceptable. In order to address these challenges, this paper proposes a set of novel improvement methods as follows. Firstly, the ORiented Brief (ORB) method is used in feature detection and descriptor extraction. Secondly, a bidirectional Fast Library for Approximate Nearest Neighbors (FLANN) k-Nearest Neighbor (KNN) algorithm is applied to feature match. Then, the improved RANdom SAmple Consensus (RANSAC) estimation method is adopted in the motion transformation. In the meantime, high precision General Iterative Closest Points (GICP) is utilized to register a point cloud in the motion transformation optimization. To improve the accuracy of SLAM, the reduced dynamic covariance scaling (DCS) algorithm is formulated as a global optimization problem under the G2O framework. The effectiveness of the improved algorithm has been verified by testing on standard data and comparing with the ground truth obtained on Freiburg University’s datasets. The Dr Robot X80 equipped with a Kinect camera is also applied in a building corridor to verify the correctness of the improved RGB-D SLAM algorithm. With the above experiments, it can be seen that the proposed algorithm achieves higher processing speed and better accuracy.

## 1. Introduction

Internet of Things (IoT) is driving innovation in an ever-growing set of application domains, such as intelligent processing for autonomous robots. For an autonomous robot one grand challenge, which triggers many requirements for the information processing algorithm, is how to sense its surrounding environment effectively. Therefore, the integration and management of the robot sensor data and the system model of the robot are very important.

In order to navigate [1] in unknown environments, mobile robots should build environment maps and estimate their own position in such maps. Solving these two problems simultaneously is called simultaneous localization and mapping (SLAM). RGB-D SLAM algorithm is divided into two parts: “front-end” and “back-end”. The “front-end” is to build a map, and the “back-end” is to optimize a map with a nonlinear error function.

### 1.1. Introduction to the “Front-End” Algorithm

In the “front-end” of the RGB-D SLAM algorithm, feature detection and descriptor extraction algorithms are used to process RGB-D images. Then, the feature matching is implemented by using the descriptor results. Finally, motion transformation is estimated and optimized from matching results.

**Figure 1:**
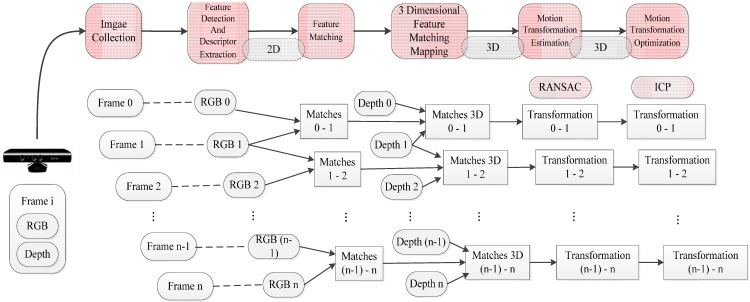
The sketch map for RGB-D SLAM algorithm in the front end.

The whole algorithm in the “front-end” is an iterative process, which is divided into feature detection, descriptor extraction, feature matching, motion estimation, and motion transformation optimization. The RGB-D SLAM algorithm in the front end is shown in Figure 1.

In iteration, two adjacent frames with RGB and depth images [2] are obtained, firstly. Then, the two RGB images are processed in the feature detection and descriptor extraction so that we can get a collection of pairs of 2D feature matching points. From each pair of 2D feature matching points, two feature points are, respectively, extracted, containing coordinate information in RGB images and depth information in depth images. After 3D coordinates in space of feature points are determined, pairs of 3D coordinate matching points are generated and then added to the collection of three-dimensional coordinates of matching point sets. According to the 3D coordinates matching point sets, the RANSAC method is used to estimate 6D motion transformation between two images. Then, 3D coordinates of matching points, ICP point cloud registration is operated by applying 6D motion transformation, after which the optimized 6D motion transformation can be obtained. The iterative process above must be repeated until there is no new inputing RGB and depth image. Therefore, the 6D motion transformation is the output of the front-end.

#### 1.1.1. Feature Detection and Descriptor Extraction

In the original RGB-D SLAM [3] descriptor algorithm, SIFT [4] and SURF algorithms are usually used to deal with the feature detection and descriptor extraction. The SIFT algorithm mainly detects features and is now widely used in the fields of computer vision and robot Visual SLAM. Whereas, the SURF algorithm has advantages on scale invariance, rotation invariance, illumination invariance, contrast invariant, and affine invariant of 64-dimensional feature descriptors. Additionally, the speed of the SURF algorithm is 3–7 times faster than that of the SIFT algorithm. Thus, the SURF algorithm is widely used the same as the SIFT algorithm in the original RGB-D SLAM.

#### 1.1.2. Feature Matching

In the original RGB-D [5] SLAM algorithm, the BruteForce method is adapted to match the feature descriptor between two successive frames. In this way, the first frame is called the search frame, and the second frame is named the training frame. In this method, feature descriptors in the search frame and training frame are complied to match. That is to say, each feature descriptor is forcibly matched with the nearest feature descriptor. There are many ways to calculate the distance, such as Euclidean distance, Manhattan distance or Hamming distance, *et al*.

#### 1.1.3. Motion Transformation Estimation

After determining the set of 3D coordinate matching points from the feature matching step, the motion transformation model of a high credibility can be estimated, according to the position relationship between the matching points. In the original RGB-D SLAM system, random sample consensus (RANSAC) is utilized. Fischler and Bolles put forward RANSAC in 1981. So far, RANSAC has been broadly used in the domain of computer vision.

#### 1.1.4. Motion Transformation Optimization

The ICP method in the original RGB-D SLAM is used to optimize the motion transformation model. It iteratively solves the rigid body transformation between two point-sets by making the distance of two point-sets minimum.

### 1.2. Introduction to the “Back-End” Algorithm

**Figure 2:**
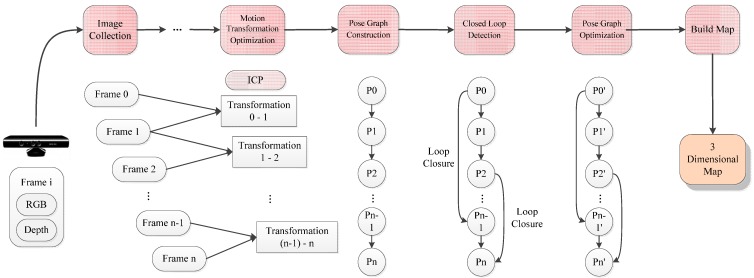
The sketch map for RGB-D SLAM algorithm in the back end.

In the back-end for RGB-D SLAM system, the graph-based pose optimization method is used, in which the initial pose graph in the front-end is used as the input data. Then, loop closure detection is performed, so false-positive loop closure constraints can be eliminated. Meanwhile, the nonlinear error function optimization method is applied to optimize the pose graph. Thus, the camera trajectory can be known after figuring out the globally-optimal camera position. The “back-end” optimization is very useful for the reconstruction of the 3D map environment. The RGB-D SLAM algorithm in the “back end” is shown in Figure 2.

Firstly, the result for the pose graph is detected for the closed-loop and is added to the closed-loop constraints. After that, the pose graph can be optimized. Therefore, pose graph nodes and edges are obtained. Meanwhile, camera pose is received before the optimization. According to different camera pose results, the point cloud will be generated, and then it is transformed to the initial position. After that, the 3D map [6] environment is reconstructed.

The rest of the paper is organized as follows: in Section 2, the related work of the SLAM based on a RGB-D sensor is given. Section 3 presents our proposed algorithm and the performance evaluations are provided in Section 4. Section 5 concludes the paper.

## 2. Related Work

There have been numerous publications of VSLAM approaches in previous years. In recent years the emergence of graph-based VSLAM [7,8,9,10] approaches has been most notably shown.

The RGB-D SLAM system was first scientifically proposed by Henry *et al.* [11] who use visual features, in combination with GICP, to create and optimize a pose graph. Unfortunately, neither the software nor the data used for evaluation has been publicly available so that a direct comparison can be carried out.

The pioneering work done by Nister *et al.* had combined feature extraction and matching with RANSAC to obtain accurate pose estimation through stereo cameras. Izadi *et al.* tackled SLAM by splitting the tracking and mapping into separate problems. In this work the camera is tracked over a global model of the environment through an ICP algorithm. The environment model is made denser and refined over several observations such that tracking is improved. Unfortunately, pure ICP relies on very structured environments, thus, limiting this technique to only a room-type scenario and not a general indoor case. Furthermore, keeping track of a dense model can be computationally expensive and will not scale very well.

Gradient histogram-based feature descriptors have been frequently used in VSLAM [12]. They are said to deliver the most descriptive features, but whose computations are expensive. Tree-based [13] algorithms can be used to find corresponding features in different images.

Henry *et al.* propose a method to jointly optimize visual features based on RANSAC with ICP. This approach is simplistic in nature but can work very intuitively with RGB-D cameras. During optimization, bundle adjustment is applied to obtain the consistent maps. Audras *et al.* argued against a feature-based method due to their uncertain nature in matching and detection by introducing an appearance-based approach [14]. Their dense stereo tracking technique [15], converted to work with the RGB-D camera, predicted relative camera motion by minimizing a warping function between frames, given 3D point cloud information.

Newcombe *et al.* proposed to incrementally build a dense model of the scene and register each new measurement to this model. Whelan *et al.* extended the KinectFusion algorithm of Newcombe *et al.* to arbitrarily large scenes. As KinectFusion does not optimize previous camera poses, there is no possibility to correct accumulated errors in this model.

Kintinuous overcomes such a limitation by virtually moving the voxel grid with the current camera pose. The parts that are shifted out of the reconstruction volume are triangulated. However, so far, the system cannot deal with loop closures and, therefore, may drift indefinitely.

This paper takes RGB-D SLAM into consideration at present, the deficiency for the original RGB-D SLAM algorithm undergoes an in-depth analysis, and each process is optimized and verified for the RGB-D SLAM one by one.

The original RGB-D SLAM algorithm’s problems can be concluded as follows.

(1) The original RGB-D SLAM algorithm runs the feature detection and descriptor extraction through the SIFT or SURF method. However, the two methods use algorithms of convolution, histogram statistics, image integration, and sliding window, which are all time-consuming strategies. Therefore, SIFT or SURF methods cannot satisfy the real-time requirement in the front-end.
(2) The original RGB-D SLAM algorithm applies BruteForce method in feature matching; there is a large error with high time complexity. When the method is used to forcibly match the search frame with the training frame in RGB-D SLAM matching, each feature descriptor will look for the minimum distance of feature descriptors in the training frames. The problems for matching are as follows:
  Firstly, the BruteForce method is matched violently, which has a high dimension of space-time complexity and easily leads to severely reducing the matching speed.
  Secondly, not every feature descriptor in the search frames can have the right matching in the training frames.
  Thirdly, when BruteForce is used, every search frame must find a fmatching point in the training frames through one direction, which leads to some error matching.
  Finally, when the same or similar objects with little difference in background appeared in the environment, the BruteForce method will regard same or similar things as the correct matching.
(3) The RANSAC method is applied to estimate the motion transformation in the original RGB-D SLAM. The evaluation data is not updated in this process and the previously evaluated data causes large errors in the assessment results.
(4) The 3D coordinate matching point is used to register the ICP point cloud in the original RGB-D SLAM. However, there is some error matching for the 3D coordinates of matching points. Therefore, in the model for the motion transformation some problems arise as follows:
  Firstly, when the point cloud is registered by the original ICP algorithm, it generates a lot of errors, such as overlap.
  Secondly, point cloud is generated by coordinates of the 3D matching points. After calculating, coordinates of the 3D matching points have some errors themselves. Therefore the motion transformation optimization model will have many errors.
  Finally, point cloud registration is utilized to optimize the motion transformation when RANSAC succeeds. However, if RANSAC fails, this procedure will be finished directly. Then the motion transformation sequence must be interrupted, which will cause large errors in building the 3D environment.

Due to the above reasons, the original RGB-D SLAM [16] algorithm appears slowly and inevitably, in the meantime, there is tremendous error in results, which cannot fulfill demands for real-time and high-precision. According to these problems, this paper proposes an improved method for the RGB-D SLAM algorithm.

## 3. Proposed Algorithms

**Figure 3:**
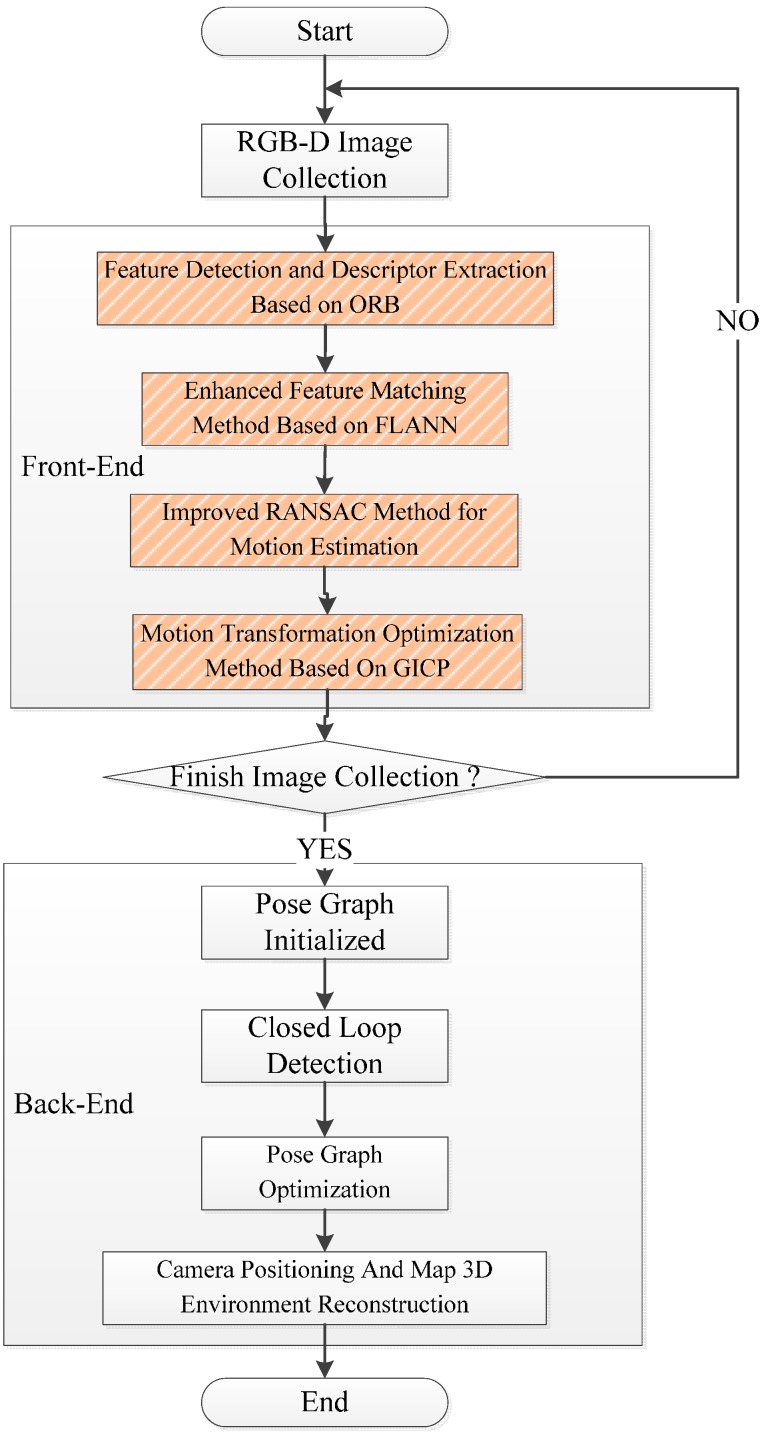
The process for improved RGB-D SLAM algorithm.

This paper proposes to use the ORB for feature detector and descriptor extraction, FLANN KNN for feature matching enhancement, and an improved RANSAC for motion transformation estimation and the optimized GICP for motion transformation. The improved RGB-D SLAM [17] has great efficiency and high precision. The process is shown in Figure 3.

The improvement contents are listed as follows:

(1) The ORB is applied to filter illegal depth information in the feature detection and descriptor extraction.
(2) The bidirectional FLANN KNN is used to improve accuracy in feature matching.
(3) The RANSAC is utilized to get more accurate inlier matching points in motion transformation estimation.
(4) The high accuracy GICP is applied to improve the speed and accuracy for point cloud registration in motion transformation.

### 3.1. The ORB Method for Feature Detection and Descriptor Extraction

The ORB is to detect features and, in the meantime, filter the illegal depth information [18]. The reason for filtering the illegal depth information is that such information can lead to large errors in results. However, when the illegal depth information was handled in feature detection, it can effectively decrease the number of descriptors, the computation [19] complexity, and also improve the speed.

### 3.2. Enhanced Feature Matching Method Based on FLANN

The process for the enhanced feature matching method based on FLANN is shown as follows:

Firstly, solving the slow speed of BruteForce problem, this paper runs the feature matching based on FLANN. The FLANN [20] is an implementation of the fast approximate nearest neighbor search library. For the nearest neighbor search in high dimensional space, FLANN has the best performance in the hierarchical search algorithm [21]. Compared with traditional nearest neighbor search algorithms, FLANN is improved by an order of magnitude.

Secondly, in order to find all the feature points of nearest distance in training frames, the search results are obtained by the FLANN KNN method and, then, are filtered by constraints. When a set $P=\{p_{1},p_{2},...,p_{n}\}$ and a query point q∈M are defined in orientation M space, the NN(q,p)∈P is the nearest neighbor elements of q which is defined as $NN(q,P)=\arg\min_{x\in p}d(x\in p)d(q,x)$, where d(q,x) expresses the distance of q and *x*. The searching method for KNN is to find nearest neighbors of *K*, which is defined as follows:

(1) KNN(q,P,K)=A

where A is satisfied as Formula (2), it shows the constraints for $P=\{p_{1},p_{2},...,p_{n}\}$ set.

(2) |A|=K,A⊆P,∀x∈A,y∈P−A,d(q,x)≤d(q,y)

In the KNN search method, let K=2, *i.e.*, every feature point $s_{i}$ in search frames finds its two nearest neighbor such as $t_{i1}$ and $t_{i2}$ in training frames, the distance of $s_{i}$ between them is $d_{i1}$ and $d_{i2}$. Compared the distance between $d_{i1}$ and $d_{i2}$, we regard $s_{i}$ and $t_{i}$ as the correct matching points when the distance of $d_{i1}$ is less than $d_{i2}$. Otherwise, when the distance for $d_{i1}$ and $d_{i2}$ is relatively close, we think that $s_{i}$ and $d_{i1}$, $s_{i}$, and $d_{i2}$ are not the correct matching points. Let $ratio_{i}=\frac{d_{i1}}{d_{i2}}$, when the ${\text{ratio}}_{i}$ is less than 0.6, we keep the corresponding matching points; otherwise, they must be removed.

Thirdly, in order to eliminate the error matching points for the single direction matching, the bidirectional FLANN KNN is used to search the correct matching points. For every feature point in search frames, the FLANN is used to find matching pairs in training frames and, meantime, it filters the nearest and the next nearest neighbor similar distance of the matching point. Then, the good matching points set of *s*_1_ can be recorded. Eventually, the above steps are executed in reverse direction and the good matching point set of *s*_2_ can be recorded.

Finally, in order to solve the same or similar objects in the environment, the homography matrix transformation is applied to optimize the matching results. The homography transformation is a projective plane to the reversible transformation of another plane. Two images in the same plane can be linked together through homography transformation. Then, the results are shown in a homography matrix. The homography matrix obtains matching point relationships between two images. In this paper, removing the error matching points is based on whether the object appeared in a scene. However, the background in frames has high similarity; therefore, we can eliminate these repeated object error matches without matching the background. In details, each pair(i,j)∈S extracts the coordinates $\left(x_{i},y_{i}\right)$ in query frames and the coordinates are added into query position sets in searching frame sets. The coordinates $\left(x_{i},y_{i}\right)$ extracted in training frames are added to training position sets. Then, the two coordinate sets are used to obtain the optimized homography transformation matrix through RANSAC. Meanwhile, the set *s* removes the matching pairs which cannot satisfy the homography matrix constraints. Finally, after all matching points are traversed by performing the above operations, the set S will have high confidence.

To sum up, the enhanced feature matching FLANN KNN method is used to match feature descriptors in the search and training frames, and the homography matrix transformation is utilized to optimize the matching results. The corresponding feature matching point set can be obtained by the improved feature matching method. After that, the depth information of two feature points is extracted from the depth information matrix, and the corresponding matching points are generated to be added into the 3D coordinates of matching point sets.

### 3.3. Improved RANSAC Method for Motion Estimation

RANSAC is an iterative process. The improved methods are as follows:

Firstly, the motion transformation model is obtained using inliers in an iterative process [22]. The entire 3D coordinates of matching points are evaluated to obtain the new pairs of inliers. The new inliers can reflect the true situation of the motion transformation model.

Secondly, the iteration process obtains the optimal motion transformation model, optimal inlier sets and some evaluated errors. If the number of local inliers is higher than the threshold value, and the errors are below the threshold, the motion transformation model should be calculated again by the optimal inliers set and recorded as a final motion transformation model. Then, the final motion transformation model is used to evaluate and calculate the final inliers set.

### 3.4. Motion Transformation Optimization Method Based On GICP

GICP is based on the standard ICP and the surface ICP, and solves the motion transformation through the probabilistic model. In order to simplify the description, we assume that two point clouds $A={\left\{a_{i}\right\}}_{i=1,...,N}$ and $B={\left\{b_{i}\right\}}_{i=1,...,N}$ are obtained after two matching points are calculated in circulation part on the standard ICP algorithm. Meanwhile, assuming the relation satisfying the condition of $||m_{i}-T\cdot b_{i}||>d_{\max}$ is deleted in A and B sets, the GICP algorithm is improved as follows:

Assuming $\hat{A}=\left\{\hat{a_{1}}\right\}$ and $\hat{B}=\left\{\hat{b_{1}}\right\}$ exist in the probabilistic model, A and B will be generated by $a_{i}\sim N(\hat{a_{i}},C_{i}^{A})$ and $b_{i}\sim N(\hat{b_{i}},C_{i}^{B})$, and $C_{i}^{A}$, $C_{i}^{B}$ are the covariance matrix to the measure point. Assuming that the elements in set A exactly corresponded that in set B, the motion transformation satisfies the equation as follows:

(3) $$\hat{b_{i}}=T*\hat{a_{i}}$$

For arbitrary rigid body transformation *T*, $d_{i}^{\left(T\right)}=b_{i}-Ta_{i}$ is defined, and an equation is established as follows:

(4) T=∑i Targmindi(T)T(CiB+TCiATT)−1di(T)

Equation (3) is the improvement method for GICP. When $C_{i}^{B}=I$ and $C_{i}^{A}=0$, Equation (4) degenerates the solving constrains as motion transformation; When $C_{i}^{B}=P_{i}^{-1}$ and $C_{i}^{A}=0$, Equation (4) degenerates T={ Targmin∑i||Pi⋅di||2}.

If used directly for the motion transformation model, the 3D coordinates must have huge errors. Therefore, the final points satisfied with inlier matching are calculated in the improved RANSAC. Due to the error matching points being removed, this paper proposes that the final inliers matching point replaces the original 3D coordinate matching point to optimize the motion transformation.

When the number of matching inliers are too few (less than 20 pairs), the algorithm for GICP will fail. However, there are three methods that can be altered on GICP: first is the number threshold of the point cloud; second is that the 3D coordinates which can be used to run the matching point cloud method on GICP; third is that the GICP can be degenerated to ICP. In this paper, three methods are tested as shown in Table 1:

**Table 1:** Different results for Insufficient number of processing method in Freiburg dataset.

Method	Reducing the Number of the GICP Threshold	Matching Point Cloud for GICP	Inliers Point Cloud for ICP
deviation	0.083326	0.002055	0.000684
Time (ms)	32.335	103.716	19.530

From the Table 1, it can be seen that reducing the number of the GICP threshold leads to the poor results. Due to all the 3D coordinate points obtaining some errors for matching, the efficiency and the accuracy of the algorithm are reduced. Therefore the second method cannot be used. The third is that the method for degenerating GICP to ICP [23] is more efficient and accurate for point cloud matching.

### 3.5. Improved DCS Algorithm for Closed Loop Detection on G2O

In the original G2O [24], the DCS algorithm is used to detect the closed loop. In the DCS algorithm, Agarwal *et al.* analyzes the objective function in Equation (5). At the time, they pay special attention on how the switch variables influence the local minimum value of the objective function. They select an edge *l_mn_* as an example. The function can be divided into two parts; one contains all edges except *mn*, and the other only contains the edge *mn*. Then, the optimization problem can be represented as follows:

(5) $$\begin{array}{c} X^{*},S^{*}=\underset{X,S}{\arg\min}\sum_{i}||d_{i}^{odo}|{|}_{\sum_{i}}^{2}+\sum_{ij\neq mn}||d_{ij}^{slc}|{|}_{\Lambda_{ij}}^{2}+\sum_{ij\neq mn}||d_{ij}^{sp}|{|}_{\Xi_{ij}}^{2}+||S_{mn}(f(x_{m},u_{mn})-x_{n})|{|}_{\Lambda_{mn}}^{2}+||1-S_{mn})|{|}_{\Xi_{mn}}^{2}\\ =\underset{X,S}{\arg\min}h(X_{ij\neq mn},S_{ij\neq mn})+s_{mn}^{2}\chi_{l_{mn}}^{2}+{\left(1-s_{mn}\right)}^{2}\phi \\ =\underset{X,S}{\arg\min}h(X_{ij\neq mn},S_{ij\neq mn})+g(X_{ij=mn},S_{ij=mn}) \end{array}$$

where $\phi =\Xi_{ij}^{-1}$, the function h(⋅) considers the error of odometry constraints, all the switch priors, and loop closure constraints, except the switch prior and constraint of edge *mn*. In turn, the function g(⋅) only considers the edge *mn*. When the objective function converges, the partial derivative with respect to $s_{mn}$ must be zero. Since the term h(⋅) doesn’t contain the variable $s_{mn}$, taking the partial derivative of the Equation (5) with respect to $s_{mn}$ means seeking the partial derivative of the function g(⋅):

(6) $$\nabla g(\cdot )=\frac{\partial g(\cdot )}{\partial s}=2s\chi_{l_{mn}}^{2}-2(1-s)\Phi =0$$

Solving the Equation (6), we can obtain:

(7) $$s=\frac{\Phi }{\chi_{l}^{2}+\Phi }$$

We analyze the behavior of Equation (7), especially the range of the fraction value. It is obvious that the value of the right side of the equal sign in Equation (7) is greater than zero. However, in theory, s∈[0,1]. Therefore, through the derivation above, when the objective function obtains the minimum value, *s* is given by

(8) $$s=\min(1,\frac{\Phi }{\chi_{l}^{2}+\Phi })$$

By analyzing the property of the error function, we derive the analytic solution for calculating the weighting factor. The scaling factor *s* individually depends on the initial error of each loop closing constraint. Our solution to this problem provides a new smaller range of the variables *s*. While the change is relatively minor, the implication to optimization is profound. In sum, our algorithm not only has the advantages on computing complexity and convergence speed as the same as DCS, but also is superior to DCS [25]. It not only reduces the complexity and the running time, but also improves the convergence speed. In the following, we prove the good performance of our algorithm through many experiments.

## 4. Performance Evaluation

For the original RGB-D SLAM [26] algorithm, large errors and lack of real-time [27,28] and high precision existed. In this paper, improved methods of feature detection and descriptor extraction, feature matching, motion estimation and optimization of motion transformation [29] in the RGB-D SLAM algorithm are proposed. This section tests, compares, and evaluates these methods to verify their correctness.

In our experiments, the algorithm is running on the hardware environment for a Lenovo notebook computer; a Lenovo IdeaPad Y471A, Inter Core i5-2410M CPU @ 2.30 GHz dual core processor, three level caches and 4 GB of memory. The system environment is Ubuntu 12.04, of which the kernel version is 3.5.0-54-generic, and the compiler is GCC 4.6.3.

In order to prove the correctness [30], SIFT, SURF_default, SURF_0, ORB_default, and ORB_10000 are tested, respectively, compared, and assessed for the improved algorithm. The methods are described as follows in Table 2:

To test the feature detection and Descriptor, feature matching, motion transformation estimation, motion change optimization and the whole front-end algorithm, we use the rgbd_dataset_freiburg1_360 dataset, which contains 754 frames for RGB and Depth images.

**Table 2:** Method Description.

Methods	Description
SIFT	The original method
SURF_default	DepthMin~DepthMax is 0~5000
SURF_0	DepthMin~DepthMax is 1000~3000, which is more rigid constraints.
ORB_default	DepthMin~DepthMax is 0~5000
ORB_10000	DepthMin~DepthMax is 1000~3000, which is more rigid constraints.

### 4.1. Feature Detection and Descriptor Extraction

Feature detection and descriptor extraction can be respectively validated by methods of SIFT, SURF_default, SURF_0, ORG_default, and ORB_10000.

#### Comparison for Testing Results of Single Image

**Figure 4:**
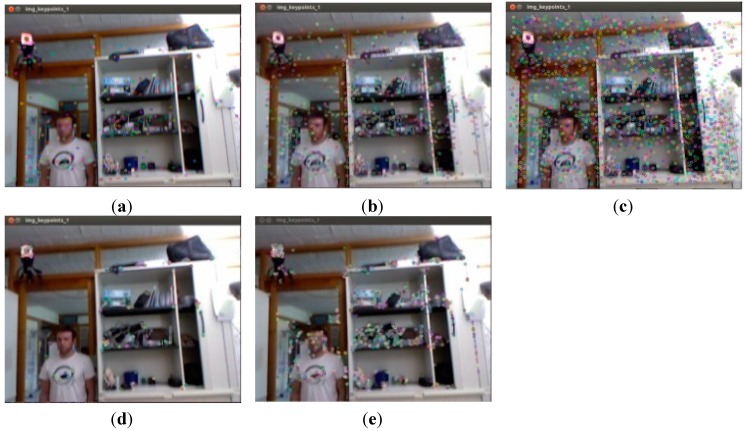
Results of 337th RGB image in datasets for feature detection and descriptor extraction (**a**) SIFT; (**b**) SURF_default; (**c**) SURF_0; (**d**) ORB_default. (**e**) ORB_10000.

In order to test feature detection and descriptor extraction, the results for 337th RGB image in datasets are shown in Figure 4.

The cost times for five methods of feature detection and feature extraction above are listed in Table 3.

From Table 3, it can be seen that cost times for ORB_default and ORB_10000 are significantly less than SIFT, SURF_default and SURF_0. However, due to the uncertainty for one image, all images in datasets should be tested to verify the correctness.

**Table 3:** Comparison cost times for five different methods.

Method for Feature Detection and Descriptor Extraction	Time for Feature Detection (ms)	Time for Descriptor Extraction (ms)	The Number for Feature Points
SIFT	34.910	49.082	350
SURF_default	100.008	169.284	1124
SURF_0	147.597	264.002	2586
ORB_default	6.622	8.302	500
ORB_10000	8.422	15.094	2462

### 4.2. Feature Matching

In original RGB-D SLAM algorithm, the matching method based on FLANN is proposed. In this section, the results for SIFT, SURF_default, SURF_0, ORB_default and ORB_10000 methods are used to validate the correctness of the improved feature matching method.

#### 4.2.1. Comparison for Two Adjacent Frames

(a) Comparison FLANN KNN with BruteForce method

**Figure 5:**
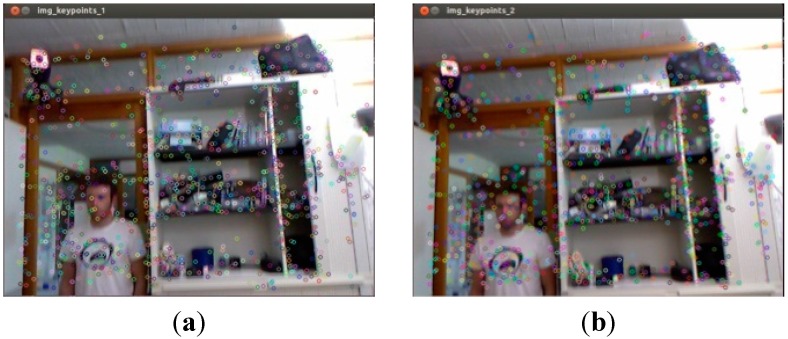
Results for SURF_default feature detection on frame 337th and 338th in datasets. (**a**) 337th Frame SURF_default; (**b**) 338th Frame SURF_default.

When BruthForce is used to feature matching, the results obtain a lot of false matching. For example, when illegal depth information feature points for 337th and 338th frames are filtered by the BruthForce method in SURF_default, results are shown in Figure 5a,b.

Assuming that features are matched by using the BruteForce method, 1124 pairs of matching points are obtained, which costs 54.725 ms. The maximum distance between the matching points is 0.780452 cm and the minimum distance is 0.025042 cm. As we can see, the maximum distance is 31 times that of the minimum. Meanwhile, there are many false matches in Figure 5a.

(b) Comparison Bidirectional KNN with KNN Results

**Figure 6:**
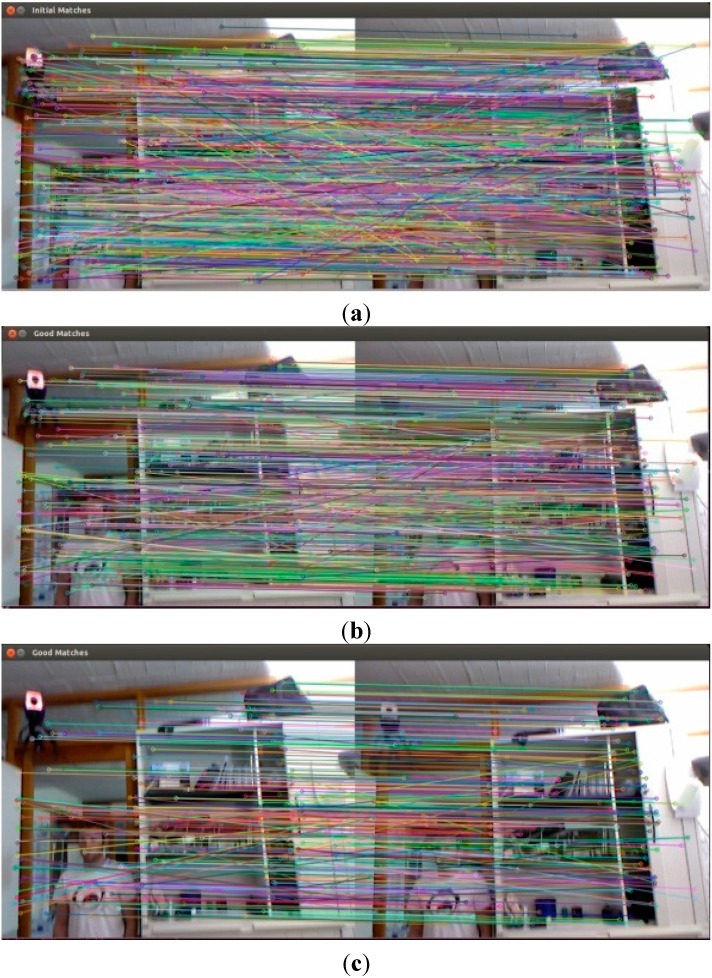

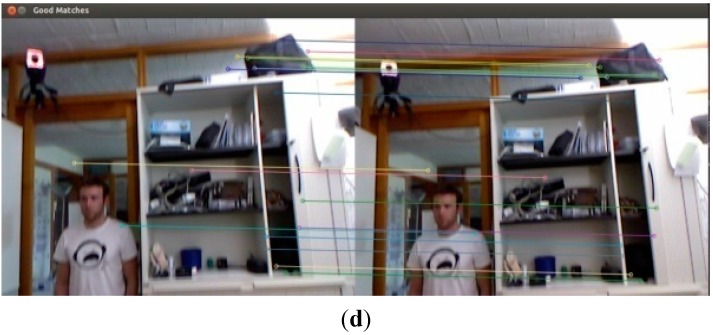
Results for BruteForce feature matching. (**a**) No threshold filtration; (**b**) 10 times the minimum distance; (**c**) five times the minimum distance; (**d**) two times the minimum distance.

As shown in Figure 6a, the FLANN KNN method still obtains some errors. Therefore, with the purpose of greater accuracy for subsequent algorithms, this paper proposes Bidirectional KNN to eliminate false matching from the FLANN KNN.

When these forward KNN matching are finished, the reverse FLANN KNN started to match. 1191 pairs of matching points are obtained, initially. The system takes 32.707 ms, the maximum distance is 0.839925 cm, and the minimum is 0.025042 cm. Then, using the nearest neighbor and next nearest neighbor ratio of 0.6 to filter, matching points are gained for 505 pairs. Wherein the maximum distance between the matching points is 0.470719 cm, the minimum distance is 0.025042 cm. The maximum distance is 18 times the minimum distance. As we can see in Figure 6b, there is less error for matching points.

**Figure 7:**
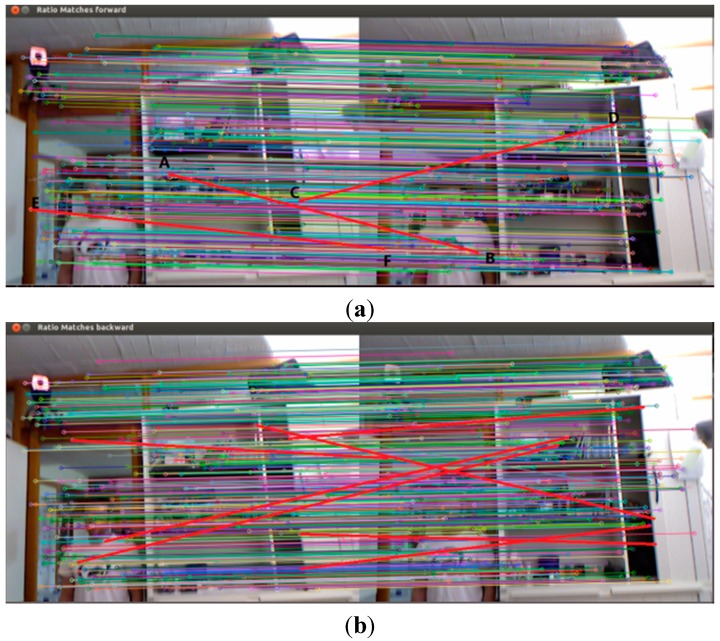

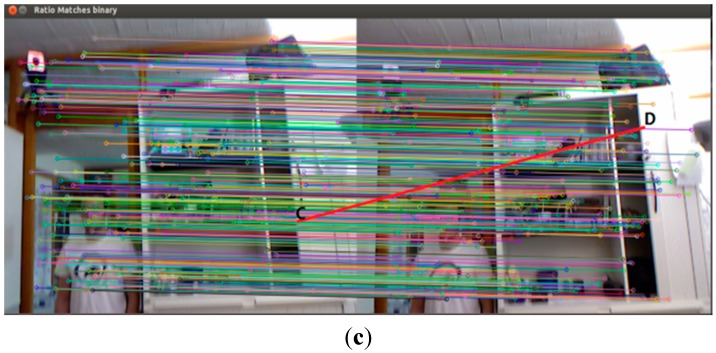
Feature matching results for Bidirectional FLANN KNN method and Homography transformation. (**a**) Forward FLANN KNN Filtration, ratio of 0.6; (**b**) Reverse FLANN KNN Filtration, ratio of 0.6; (**c**) Bidirectional FLANN KNN Filtration, ratio of 0.6.

From the above results, the forward and reverse matching results are not the same and these two matches both have some false matches. Therefore, the intersection between two matches acquires the bidirectional matching, which can eliminate false matches. The results for bidirectional matching contain 412 pairs of matching points. The maximum distance between matching pairs is 0.386766 cm, the minimum distance is 0.025042 cm, and the maximum distance is 15 times the minimum to further eliminate some false matching points, as shown in Figure 7c. Comparing Figure 7a with Figure 7b, AB and EF mismatches have been eliminated by bidirectional matching methods.

#### 4.2.2. Comprehensive Comparison for Feature Detection, Feature Extraction and Feature Matching

The total time for feature detection, descriptor extraction and feature matching is analyzed in Table 4:

**Table 4:** Comprehensive Comparison for Feature Detection, Feature Extraction and Feature Matching.

Feature Detection and Descriptor Extraction Method	Number for Feature Points	Time for Feature Detection and Descriptor Extraction (ms)	Number for Matching Pairs	Time for Feature Matching (ms)	Total Time (ms)
SIFT	168	65	83	10	140
SURF_default	604	166	182	35	367
SURF_0	2360	375	255	134	884
ORB_default	388	12	113	13	37
ORB_10000	1112	16	327	39	71

From Table 4 it can be seen that the methods which are the improved feature detection, descriptor extraction, and bidirectional FLANN KNN and homography transformation are faster than before, just 37 ms. In this case, the number of matching points is 113. The second one is ORB_10000, which took 71 ms and the number of matching pairs is 327. The third one is SIFT, which took 140 ms and the number of matching pairs is 327. The forth one is SURF_default, which took 367 ms and the number of matching pairs is 182. The last one is SURF_0, which took 884 ms and the number of matching pairs is 255. In a word, the ORB method for feature detection, descriptor extraction, and improved bidirectional FLANN KNN and homography transformation cannot only get more correct matching points, but also greatly reduces the time required for the algorithm.

### 4.3. Motion Transformation Estimation

#### 4.3.1. Comparison of Results to Two Adjacent Frames on Motion Transformation Estimation

Feature detection and descriptor extraction are also handled by SIFT, SURF_default, SURF_0, ORB_default, and ORB_0 on the dataset of the 337th and 338th frames. Then, for feature matching results, the number of inliers is obtained by the original and improved motion transformation estimation methods. Additionally, the proportion of inliers in pairs of matching features, the error value, and time consumption are calculated. The results are shown in Table 5.

**Table 5:** Results for motion transformation estimation on 337th and 338th frames.

Methods	The Original Motion Transformation Estimation Method			The Improved Motion Transformation Estimation Method		
	The Number of Inliers	The Proportion of Inliers (%)	Deviation	The Number of Inliers	The Proportion of Inliers (%)	Deviation
SIFT	187	77	0.0217976	134	88	0.0210702
SURF_default	622	64	0.0235837	311	91	0.022127
SURF_0	Failure	Failure	Failure	377	91	0.0218584
ORB_default	360	79	0.0250409	150	81	0.0261754
ORB_10000	1621	75	0.0221271	731	88	0.0200384

From Table 5, the inliers ratio, the error, and time consumption for improved motion transformation estimation is significantly lower than the original method. Therefore, it is obvious that improved motion transformation estimation is better than the original.

#### 4.3.2. Comparison of Motion Transformation Estimation Results for All Images in Datasets

**Figure 8:**
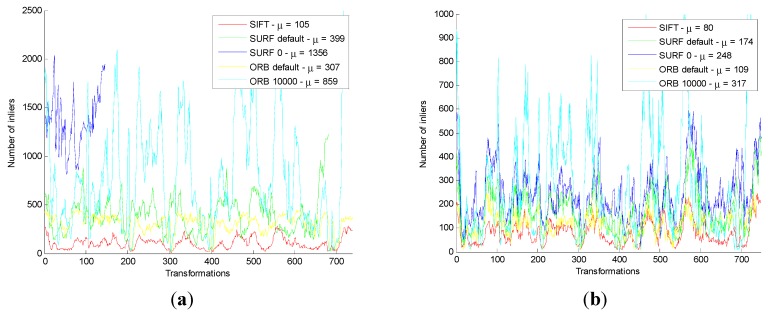

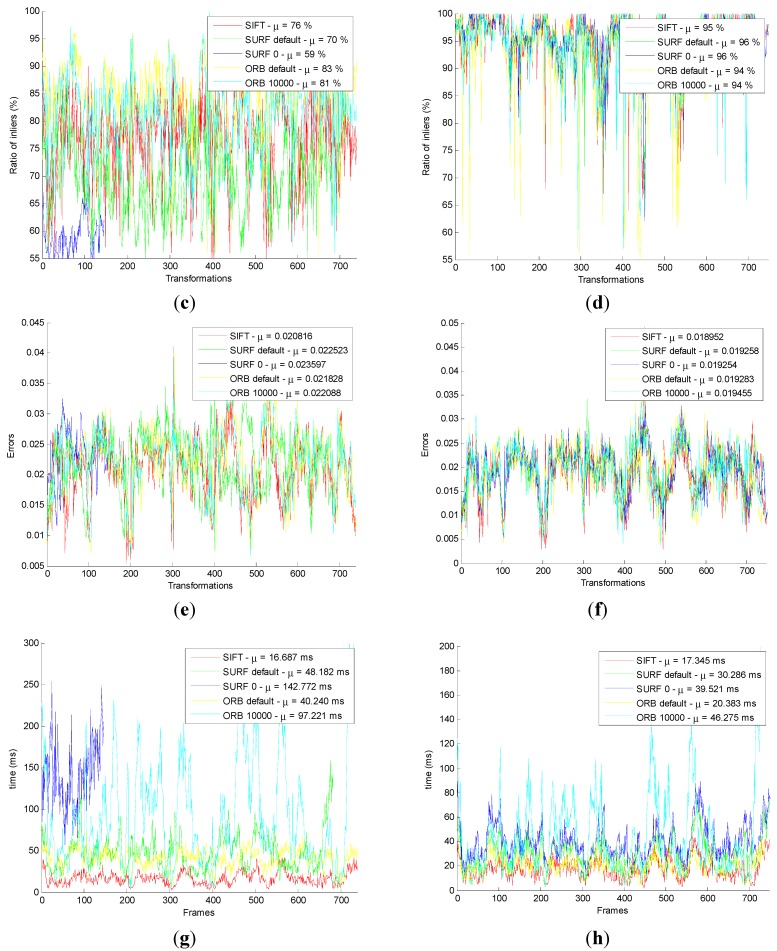
Results of motion transformation estimation for original and improved method on all images. (**a**) The number of original inliers. (**b**) The number of improved inliers. (**c**) The proportion of original inliers. (**d**) The proportion of improved inliers. (**e**) Deviation for original method. (**f**) Deviation for improved method. (**g**) Time for original method. (**h**) Time for improved method.

For all Images in datasets, the error value and consuming time are shown in Figure 8.

In Figure 8a,c,e,g, the blue line of SURF_0 is very short. The reason is that, in the original algorithm, the SURF_0 has a large number of failures in the motion transformation estimation stage. Moreover, when failure happens, the number of the corresponding inliers, the proportion, the error value, and the time for inliers on the original method are not statistical. It indicates that the algorithm is not stable. However, in Figure 8b,d,f,h, the blue line of SURF_0 is normal. It shows that the failure can be solved by the improved method.

Comparing Figure 8a with Figure 8b, whatever the feature detection and descriptor extraction method is used, the number of inliers is obviously less than the original method. Other comparisons have reached the same conclusion.

### 4.4. Motion Change Optimization

#### 4.4.1. Comparison Results of Motion Change Optimization for Adjective Frames

The original and the improved method deal with motion transformation optimization by 337th and 338th frame images in datasets. The error value and time consumption are shown in Table 6:

**Table 6:** Results for motion transformation optimization method.

Method	The Original Motion Transformation Optimization Method		The Improved Motion Transformation Optimization Method	
	Deviation	Time (ms)	Deviation	Time (ms)
SIFT	0.00444218	19	0.000964601	59
SURF_default	0.00397572	51	0.000806677	87
SURF_0	failure	failure	0.000776322	97
ORB_default	0.0113304	36	0.000661158	49
ORB_10000	0.00254392	101	0.00046986	354

From Table 6, even though the improved motion transformation optimization spends 1.5–3 times for the original method, the error is smaller by one order of magnitude than the original method, and it can deal with failure of the original method. Therefore, the improved motion transformation optimization method is correct.

#### 4.4.2. Comparison Results of Motion Transformation Optimization for All Images in Datasets

**Figure 9:**
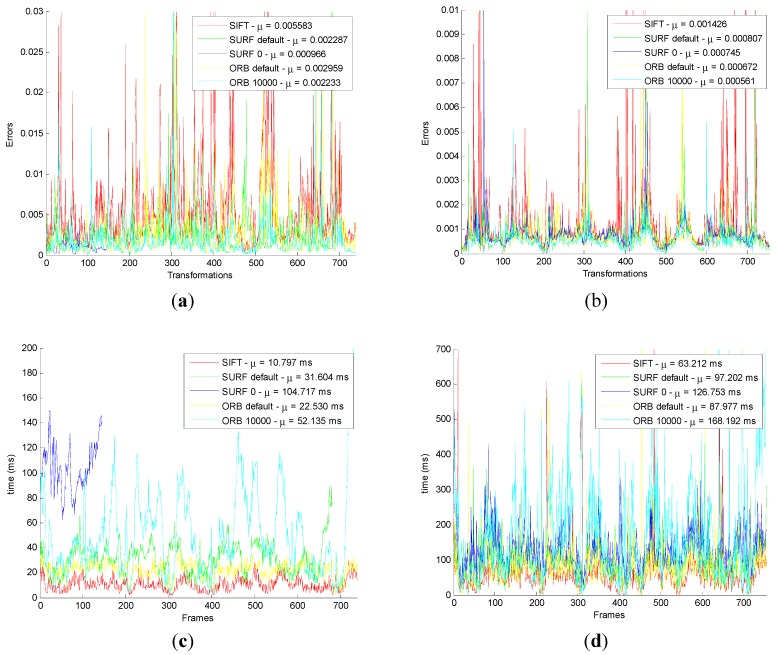
Comparison results of motion transformation optimization for all images in datasets. (**a**) The original error; (**b**) The improved error; (**c**) The original time consumption; (**d**) The improved time consumption.

The original and improved motion transformation optimization method are tested by 753 adjacent frames in Freiburg 360 datasets, and obtained the error value and time, as shown in Figure 9.

After comparison Figure 9a with Figure 9b, it can be found that the error of the improved method is one order of magnitude smaller than the original motion transformation optimization. From Figure 9c,d, the time for the improved method is significantly less than the original motion transformation optimization. Even though the time is longer than ICP in a certain extent, the accuracy of the GICP algorithm is much higher than that of ICP. In order to improve the accuracy of the algorithm, a little time can be acceptable.

In Figure 9c the blue line of SURF_0 is very short. The reason is that in the original algorithm, SURF_0, has a large number of failures in the motion transformation estimation; the line is shorter, the failures are more numerous. This indicates that the unstable algorithm needs to be improved. However, in Figure 9d, the blue line of SURF_0 is normal. This shows that the failure can be solved by the improved method.

### 4.5. Comparison of the Whole “Front-End” Algorithm

Firstly, the original SIFT, SURF_default, SURF_0, ORB_default, and ORB_10000 methods are used in the original front-end algorithm, which include feature detection, feature descriptor, and original feature matching. After that, using the original motion transformation estimation method obtains the motion transformation.

Then, the original and the improved five motion transformation are compared with each other. The results are shown below.

#### Comparison of the “Front-End” Optimization Results with Ground Truth in Datasets

In order to prove the correctness and effectiveness, the whole algorithm is compared and evaluated by the translational error and rotational error.

**Figure 10:**
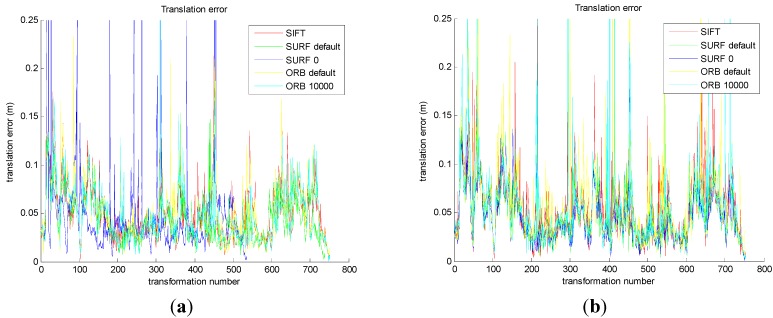

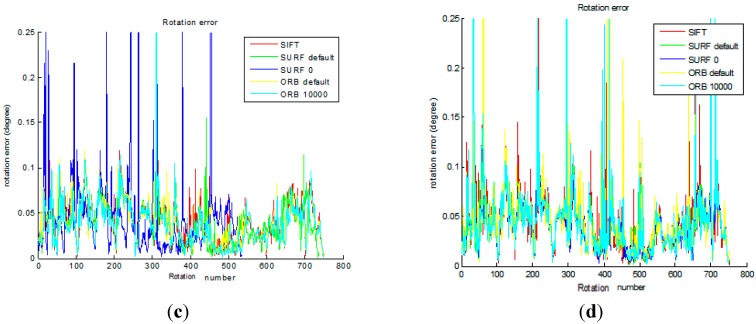
Comparison of the original and the improved algorithm for transformational error and rotational error. (**a**) The transformational error in original; (**b**) The transformational error in improved; (**c**) The rotational error in original; (**d**) The rotational error in improved.

The results are shown in Figure 10a,c. Then, comparing the improved five groups of the motion transformation sequence with ground truth in datasets, the translational error and rotational error of the five methods are calculated, respectively. The results are shown in Figure 10b,d.

Figure 10a shows that the five translational errors are obtained from the original front-end algorithm. As we can see, the translational errors of the five methods are similar, which is about 0.05 m. Figure 10b shows that the five translational errors are obtained from the improved front-end algorithm. And the translational errors of the five methods are similar, which is about 0.05 m. Figure 10c shows that the five rotational errors are obtained from the original front-end algorithm. As we can see, the rotational errors of the five methods are similar, which is about 0.05°. Figure 10d shows that the five rotational errors are obtained from the improved front end algorithm. As we can see, the rotational errors of the five methods are similar, which is about 0.05°.

From Figure 10a,b, compared with the original method, the improved method decreases the translational error in SURF_0 and ORB_10000. From Figure 10c,d, the improved method decreases the rotational error in SURF_0 and ORB_10000, compared with the original method. It is important to note that the translational error staying around 0.05 m and rotational error being 0.05° depend on the inherent error of Kinect camera.

(1) Comparison of Camera Pose In “Front-End”

Camera pose can be used to prove the correctness and effectiveness in five methods from the original and improved “front-end” algorithm.

**Figure 11:**
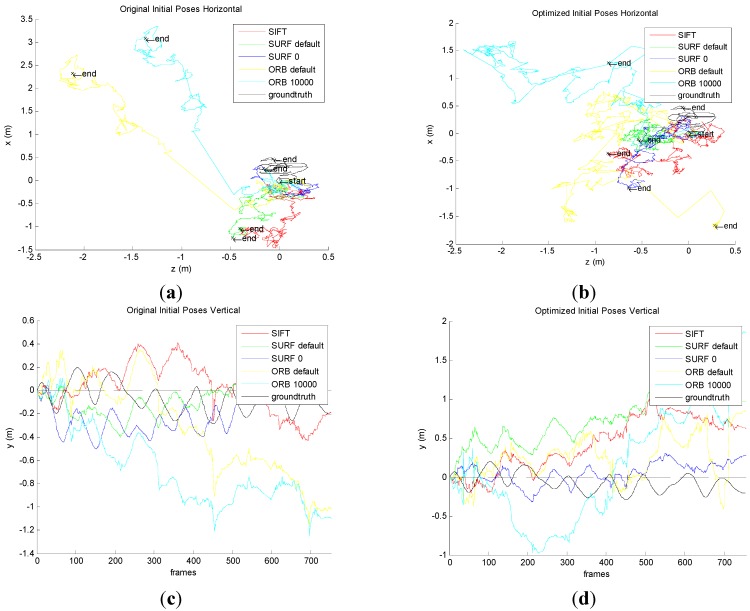
Comparison the original and the improved for Camera pose. (**a**) Horizontal poses for original algorithm; (**b**) Horizontal poses for improved algorithm; (**c**) Vertical poses for original algorithm; (**d**) Vertical poses for improved algorithm.

Firstly, camera poses are obtained by the original five groups of the motion transformation sequence, and the real camera poses are received by searching ground truth. The results are shown in Figure 11a,c. Then, camera poses are obtained by the improved five groups of the motion transformation sequence, and the real camera poses are received by searching ground truth. The results are shown in Figure 11b,d.

Figure 11a shows that the original front-end algorithm obtains the camera pose in horizontal, which is the *X* axis and *Z* axis of the pose. No matter which method in the original front-end algorithm is used, it must have errors compared with the ground truth. We can conclude that the minimum error is the method of SURF_0. The results of SIFT and SURF_default are slightly larger, and that of ORB_default and ORB_10000 is much larger.

Figure 11b shows that the improved front-end algorithm acquires the camera pose in horizontal, which is the *X* axis and *Z* axis of the pose. No matter which method the original front-end algorithm of method is used, it has errors compared with the ground truth. We can conclude that the minimum error is the method of SURF_0. The results for SIFT and SURF_default are slightly larger, and the ORB_default and ORB_10000 is much larger. However, it has obvious improvements compared to the original algorithm.

Figure 11c shows that the original front-end algorithm obtains vertical posture which is the *Y* axis of the pose. No matter which method the original front-end algorithm of method is used, it must have errors compared with the ground truth. Smaller errors are seen in SIFT, SURF_default and SURF_0. The results for ORB_default and ORB_10000 are much larger.

Figure 11d shows that the improved front-end algorithm acquires vertical posture which is the *Y* axis of the pose. No matter which method of the improved front-end algorithm is used, it must have errors compared with the ground truth. However, compared with the original front-end algorithm, it has been greatly improved.

From the comparison for four pictures, the improved algorithm reduces the error for camera pose, which proved the correctness.

(2) The Results for Stitching On “Front-End” Map

Five methods for SIFT, SURF_default, SURF_0, ORB_default, and ORB_10000 are, respectively, compared by the original and improved algorithm.

**Figure 12:**
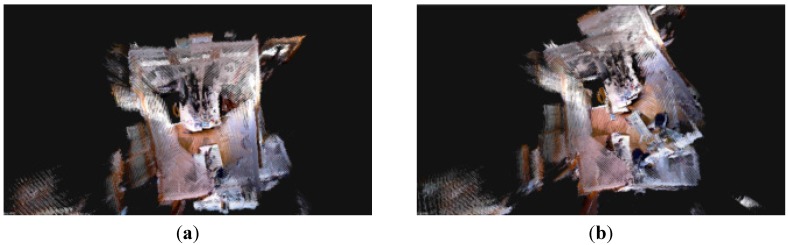

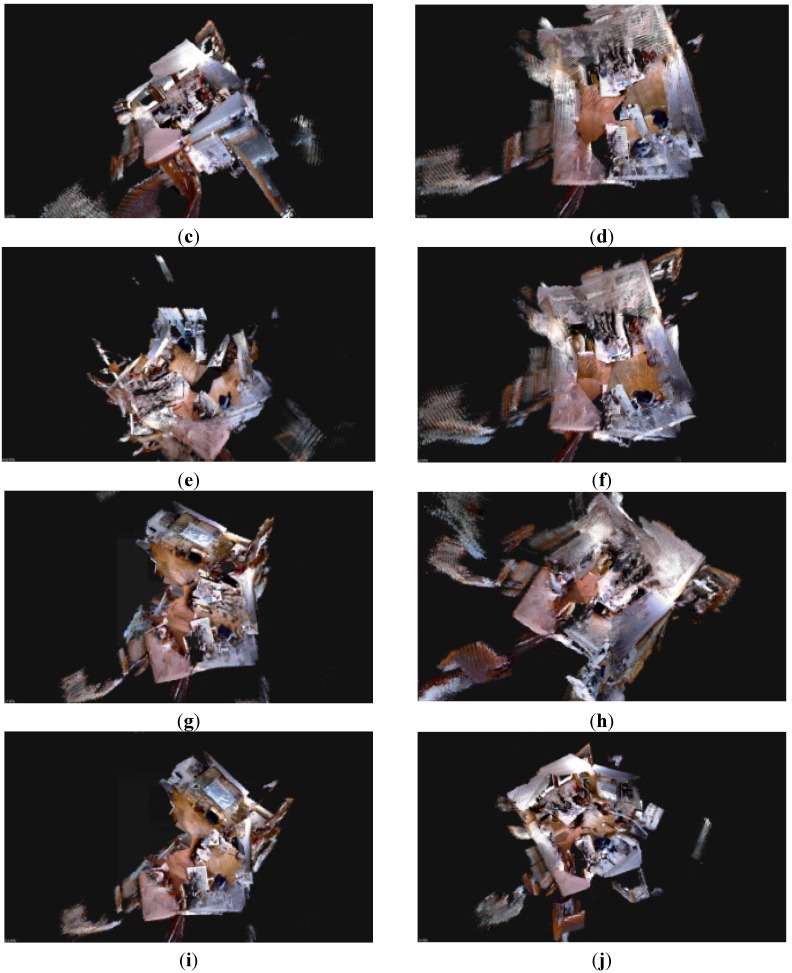
The results for stitching on the original and the improved front-end map. (**a**) Original SIFT; (**b**) Improved SIFT front end; (**c**) Original SURF_default; (**d**) Improved SURF_default; (**e**) Original SURF_0; (**f**) Improved SURF_0; (**g**) Original ORB _default; (**h**) Improved ORB _default; (**i**) Original ORB _10000; (**j**) Improved ORB _10000.

First, the original front-end algorithm is implemented by SIFT, SURF_default, SURF_0, ORB_default, and ORB_100. Then, according to the front-end algorithm, the maps that the camera poses, joined, are shown in Figure 12a,c,e,g,i. Meanwhile, the improved front-end algorithm is implemented by SIFT, SURF_default, SURF_0, ORB_default, and ORB_100, after that, the map of the camera poses, joined, are shown in Figure 12b,d,f,h,j.

Comparing (a), (c), (e), (g), and (i) in Figure 12, the horizontal and vertical error for SIFT and SURF_default in the front-end algorithm is smaller. However, the error from SURF_0, ORB_default, and ORB_10000 is very large, with serious bending directly occurring in stitching results.

Comparing (b), (d), (f), (h), and (j) in Figure 12, splicing errors from the five methods is small. The splicing results for SURF_0, ORB_default, and ORB_10000 method improves, obviously, which proved the correctness of the improved front-end algorithm.

### 4.6. The Actual Environment Test Based on Dr Robot X80

From the above experiments, we use the algorithm in the real Dr Robot X80 environment to verify its effectiveness.

**Figure 13:**
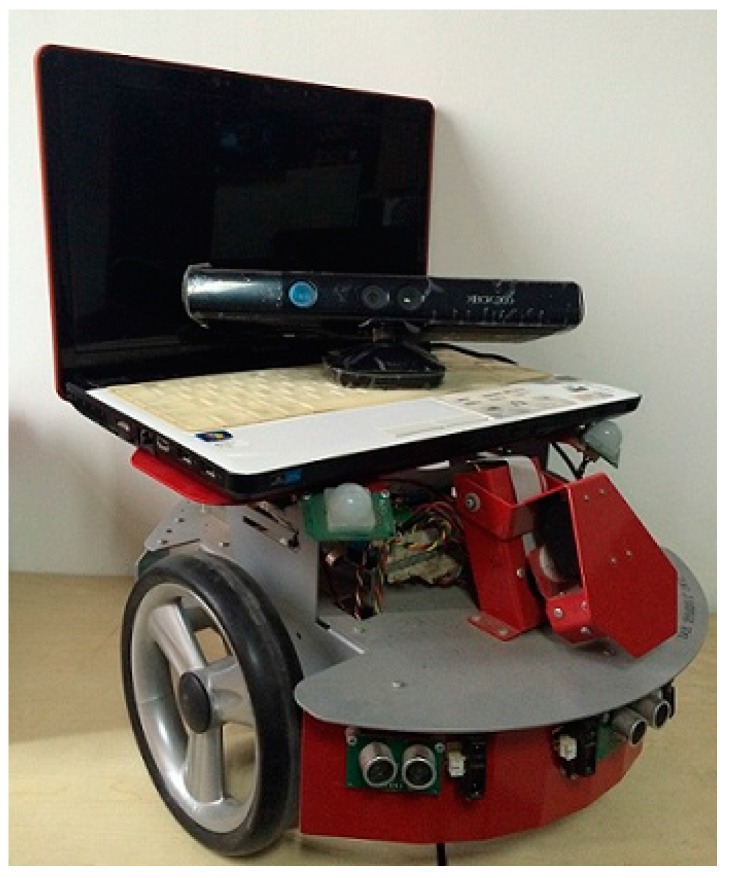
RGB-D SLAM system architecture based on Dr Robot X80.

RGB-D SLAM system architecture based on Dr Robot X80 is shown in Figure 13:

The RGB-D SLAM system for Dr X80 Robot is built in the environment above, and collects 475 RGB-D images. Then, SIFT, SURF, and ORB are used to test the original and improved RGB-D SLAM algorithm by the 475 frames of RGB-D images.

First, the original RGB-D SLAM algorithm uses SIFT, SURF, and ORB are run. The splicing results of a few feature points for the original front-end algorithm are poor, and there is no effective optimization for the front-end results.

**Figure 14:**
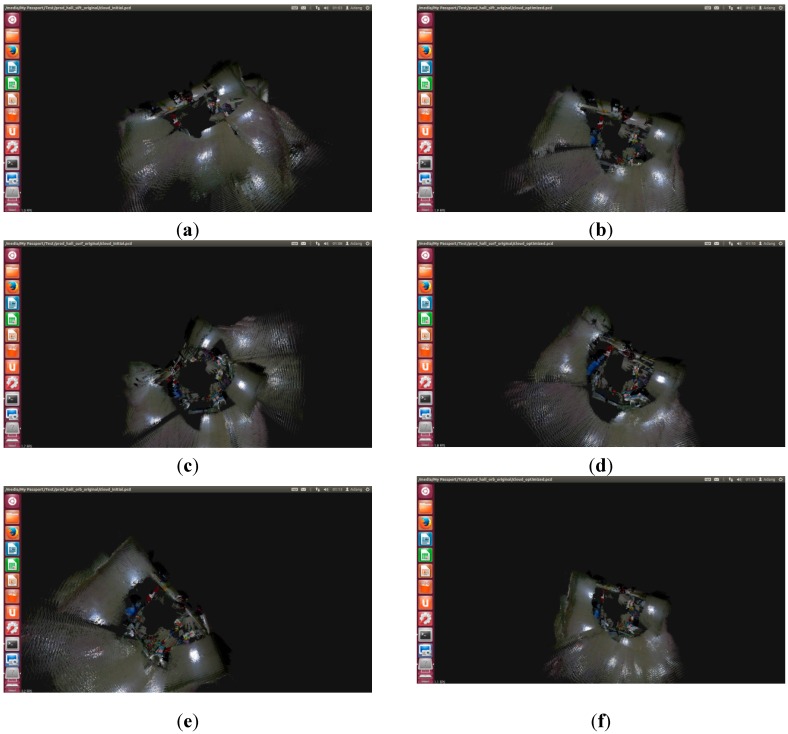
The splicing results for the original RGB-D SLAM based on Dr Robot X80. (**a**) SIFT for the front-end; (**b**) SIFT for the back-end; (**c**) SURF for the front-end; (**d**) SURF for the back-end; (**e**) ORB for the front-end; (**f**) ORB for the back-end.

Then, SIFT, SURF, and ORB are used in the improved RGB-D SLAM algorithm. The front-end results are shown in Figure 14a,c,e. The improved method has very good results and a small error. The back-end results are shown in Figure 14b,d,f. The improved RGB-D SLAM back-end algorithm has better splicing results in the case of less feature points.

**Figure 15:**
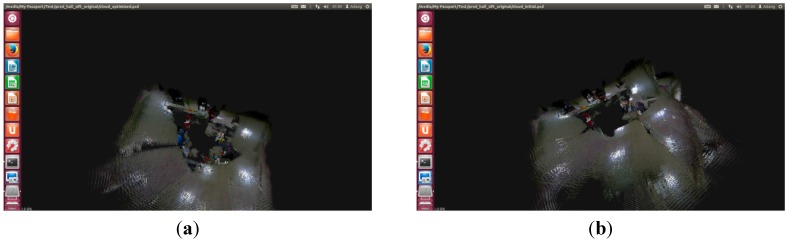

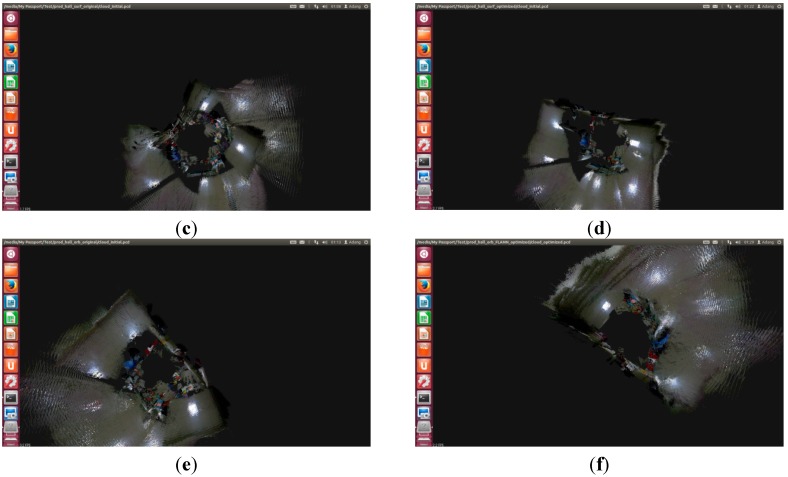
The splicing results for the improved RGB-D SLAM based on Dr Robot X80. (**a**) SIFT for the front-end; (**b**) SIFT for the back-end; (**c**) SURF for the front-end; (**d**) SURF for the back-end; (**e**) ORB for the front-end; (**f**) ORB for the back-end.

**Figure 16:**
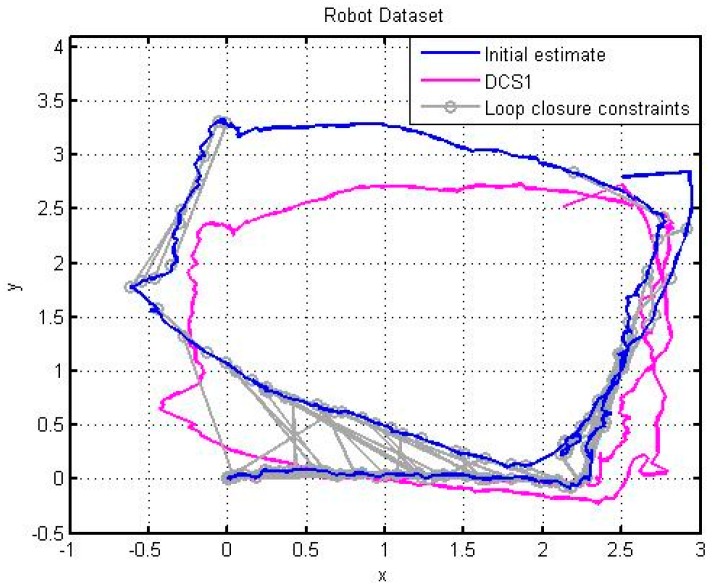
The optimization of Robot trajectory by the improved RGB-D SLAM based on Dr Robot X80.

Comparing Figure 14 with Figure 15, the improved RGB-D SLAM algorithm not only has better spliced the 3D map, but also obtains the accurate trajectory of the robot. The trajectory for the robot is shown in Figure 16.

Thus, the improved RGB-D SLAM algorithm can successfully solve the splicing problem for the few feature points.

In summary, through a lot of experiments, this chapter evaluates the efficiency and accuracy for the improved RGB-D SLAM algorithm and verifies the correctness for each step.

## 5. Conclusions and Outlook

In order to clearly illustrate the results for above sections, the names of different methods are defined in Table 7.

**Table 7:** The name for method definition table.

Method Name	The Special Content
Original SIFT_RGB-D_SLAM	Original SIFT+ Original matching + Original RANSAC+ Original ICP+ Original back-end
Original SURF_RGB-D_SLAM	Original SURF+ Original matching + Original RANSAC+ Original ICP+ Original back-end
Original ORB_RGB-D_SLAM	Original ORB+ Original matching + Original RANSAC+ Original ICP+ Original back-end
Improved SIFT_RGB-D_SLAM	Improved SIFT+ Improved matching + Improved RANSAC+ Improved ICP+ Original back-end
Improved SURF_RGB-D_SLAM	Improved SURF+ Improved matching + Improved RANSAC+ Improved ICP+ Original back-end
Improved ORB_RGB-D_SLAM	Improved ORB+ Improved matching + Improved RANSAC+ Improved ICP+ Original back-end

The implementation of the results before can be easily seen as Table 8.

**Table 8:** Schematic diagram for a comprehensive analysis of the experimental results.

		Speed	
		Low	High
Precision	Low	Original SIFT_RGB-D_SLAM, Original SURF_RGB-D_SLAM	Original ORB_RGB-D_SLAM
	High	Improved SIFT_RGB-D_SLAM, Improved SURF_RGB-D_SLAM	Improved ORB_RGB-D_SLAM

As shown in Table 8, the red part is the RGB-D SLAM algorithm with low speed and low precision; the green part is the RGB-D SLAM algorithm with high speed and high precision; the orange part for the upper left corner is the RGB-D SLAM algorithm with low speed and high precision; the orange part for the lower left corner is the RGB-D SLAM algorithm with high speed and low precision.

Both the original SIFT_RGB-D_SLAM algorithm and the original SURF_RGB-D_SLAM algorithm are slow in speed and low in precision. The speed and accuracy of the original SIFT_RGB-D_SLAM algorithm are slightly higher than the original SURF_RGB-D_SLAM algorithm, and the original ORB_RGB-D_SLAM algorithm is high in speed and low in accuracy. The improved SIFT_RGB-D_SLAM algorithm and improved SURF_RGB-D_SLAM algorithm is a low speed and high precision algorithm, the improved SIFT_RGB-D_SLAM algorithm is slightly higher than the improved SURF_RGB-D_SLAM algorithm. Compared with the original SIFT_RGB-D_SLAM algorithm, the SIFT_RGB-D_SLAM algorithm can improve the accuracy of velocity, but is slightly decreased; compared with the original SURF_RGB-D_SLAM algorithm, accuracy of the improved SURF_RGB-D_SLAM algorithm is greatly improved, and its speed is also increased slightly. The improved ORB_RGB-D_SLAM algorithm is a high speed and high precision algorithm; compared with the original ORB_RGB-D_SLAM algorithm, the improved ORB_RGB-D_SLAM algorithm’s accuracy has been greatly increased, and the velocity is decreased slightly. On the other hand, accuracy of the improved ORB_RGB-D_SLAM algorithm and that of improved SIFT_RGB-D_SLAM algorithm are basically the same, but the speed of the former is much higher than that of the latter.

To sum up, the improved algorithm can solve the low speed and low accuracy problem of the RGB-D SLAM algorithm, and it satisfies the requirements of high speed and high precision. Therefore, we can conveniently apply this improved robot identification and mapping algorithm to the IoT system constructed by a robot, it can improve the robot’s automation ability, and enhance the IoT system’s robustness in real-time.
